# Wnt5a regulates dental follicle stem/progenitor cells of the periodontium

**DOI:** 10.1186/scrt525

**Published:** 2014-12-15

**Authors:** Lusai Xiang, Mo Chen, Ling He, Bin Cai, Yu Du, Xinchun Zhang, Chen Zhou, Chenglin Wang, Jeremy J Mao, Junqi Ling

**Affiliations:** Guanghua School of Stomatology, Hospital of Stomatology, Sun Yat-sen University, Guangdong Key Research Laboratory, Guangzhou, 510055 China; Columbia University Medical Center, Center for Craniofacial Regeneration, 630 West 168 Street – PH7 East CDM, New York, NY 10032 USA

## Abstract

**Introduction:**

Dental follicle gives rise to one or several tissues of the periodontium including the periodontal ligament, cementum and/or alveolar bone. Whether Wnt5a is expressed in the postnatal periodontium or regulates dental follicle stem/progenitor cells is unknown.

**Methods:**

Dental follicle stem/progenitor cells were isolated from postnatal day 1 (p1) to p11 from rat mandibular first molars. Immunolocalization mapped Wnt5a expression in the alveolar bone, periodontal ligament, and the developing ameloblast and odontoblast layers. Mononucleated and adherent cells were isolated from p7 dental follicle. Wnt5a was overexpressed in dental follicle stem/progenitor cells to study their proliferation, osteogenic differentiation and migration behavior, with subpopulations of native dental follicle stem/progenitor cells as controls, using real-time PCR (Taqman), Lenti-viral transfection, Western blotting and immunofluorescence.

**Results:**

Wnt5a was expressed consistently in p1 to p11 rat peridontium. Native, p7 dental follicle stem/progenitor cells had modest ability to mineralize in the tested 14 days. Even in chemically defined osteogenesis medium, dental follicle stem/progenitor cells only showed modest mineralization. Upon addition of 300 ng/mL Wnt5a protein in osteogenesis medium, dental follicle stem/progenitor cells displayed mineralization that was still unremarkable. Chemically induced or Wnt5a-induced mineralization of dental follicle cells only occurred sparsely. Combination of Wnt5a with 100 ng/mL BMP2 finally prompted dental follicle stem/progenitor cells to produce robust mineralization with elevated expression of Runx2, alkaline phosphatase, collagen 1α1 and osteocalcin. Thus, native dental follicle stem/progenitor cells or some of their fractions may be somewhat modest in mineralization. Strikingly, Wnt5a protein significantly augmented RANKL ligand, suggesting putative regulatory roles of dental follicle stem/progenitor cells for the monocyte/osteoclast lineage and potential involvement in alveolar bone remodeling and/or resorption. P-Jnk1/2 was activated in Wnt5a overexpressed dental follicle cells; conversely, exposure to SP600125, a c-Jun N-terminal kinase (JNK) inhibitor attenuated Runx2, collagen 1α1 and osteocalcin expression either in the presence or absence of Wnt5a. Wnt5a overexpression in dental follicle stem/progenitor cells significantly reduced their proliferation rates, but robustly augmented their migration capacity.

**Conclusions:**

These findings provide a glimpse of Wnt5a’s putative roles in dental follicle stem/progenitor cells and the periodontium with implications in periodontal disease, tooth eruption, dental implant bone healing and orthodontic tooth movement.

## Introduction

Dental follicle stem/progenitor cells (DFSCs) develop into one or several components of the periodontium including the periodontal ligament (PDL), cementum and/or alveolar bone, all of which have potential implications in periodontal disease, tooth eruption, orthodontic tooth movement and dental implant bone healing. How DFSCs differentiate into unmineralized PDL or mineralized alveolar bone or cementum is poorly understood. Wnt signaling has been shown recently to play significant roles in tooth development, and yet in ways that are only fragmentally understood [1, 2]. Unlike classic canonical Wnt/β-catenin signaling, Wnt5a acts via the noncanonical Wnt pathway and has only been sparsely investigated in tooth development. Previous work has shown Wnt5a expression in dental papilla and enamel knot in E14.5 and E16.5 tooth germs [3, 4], as well as primarily in dental papilla of 2-month to 3-month embryonic human tooth germs [5]. Wnt5a mutant mice showed disturbed cusp formation, and delayed eruption [4, 6], suggesting Wnt5a's involvement in tooth crown and root development. However, little is known of Wnt5a expression in DFSCs that differentiate into the periodontium or whether Wnt5a plays important roles in postnatal dental follicle development.

Tooth eruption is inseparable from the growth and modeling of alveolar bone. Wnt5a plays crucial roles in bone apposition and osteoclastogenesis [7, 8]. Wnt5a acts via a noncanonical Wnt pathway through tyrosine kinase-like orphan receptor (Ror) proteins [9]. Osteoblast-lineage cells express Wnt5a, while osteoclast precursors express Ror2 [8]. The roles of Wnt5a in osteoclastogenesis are potentially related to tooth eruption and alveolar bone remodeling in periodontal diseases, although little experimental evidence currently exists in support of these putative roles. Wnt-5a activates Nemo-like kinase, which in turn phosphorylates a histone methyl transferase, leading to a co-repressor complex that inactivates PPARγ function, suggesting PPARγ suppression in favor of osteoblastic differentiation from mesenchymal stem/stromal cells via noncanonical Wnt signaling [10]. Despite the improved understanding of Wnt5a involvement in bone development and homeostasis, little is known about the roles of Wnt5a in the periodontium, one of the presumptive derivatives of DFSCs that develop into not only the PDL but also alveolar bone and cementum. The objective of the present study was to investigate Wnt5a expression in postnatal dental follicle and its roles in the proliferation, migration and differentiation of DFSCs.

## Methods

### Samples and immunohistochemistry

Following animal ethics approval by Sun Yat-sen University Medical Center, Sprague–Dawley rats were sacrificed on postnatal days 1, 3, 5, 7, 9 and 11. The mandible was resected *en masse*, immediately fixed in 4% paraformaldehyde at 4°C overnight and then transferred to ethylenediamine tetraacetic acid at 4 °C for 5 days. Following graded alcohol dehydration and paraffin embedding, the mandible was cut sagittally into 5 μm thickness sections. Anti-Wnt5a (1:50; Abcam, Cambridge, MA, USA) was used for immunohistochemistry with streptavidin–biotin peroxidase complex. The negative controls were incubated with phosphate-buffered saline in the absence of primary anti-Wnt5a antibody. Immunohistochemical methods followed our prior work [11–13].

### Isolation and culture of dental follicle stem/progenitor cells

**Figure 1:**
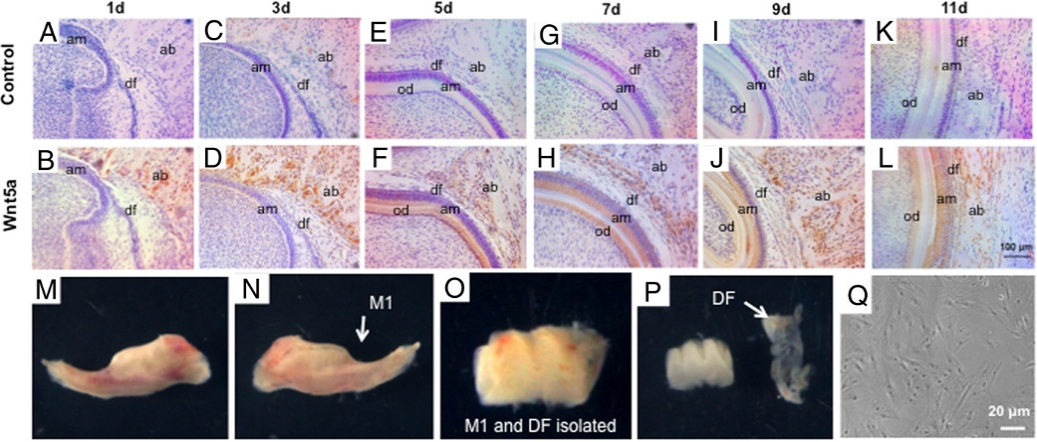
**Wnt5a expression in dental follicle and cell isolation.** Wnt5a was immunolocalized in postnatal day 1 to 11 tooth germs **(B, D, F, H, J, L)** with controls (no primary antibodies) **(A, C, E, G, I, K)** by immunohistochemistry. am, ameloblasts; od, odontoblasts; df, dental follicle; ab, alveolar bone. **(M, N, O, P)** Isolation of dental follicle from 7-day-old rat tooth germ. M1, first molar; DF, dental follicle. **(Q)** Isolated dental follicle cells plated.

Dental follicles of 7-day-old Sprague–Dawley rats were carefully isolated from the mandibular first molar tooth germs under dissection microscope (Figure 1M,N,O,P) as per our prior methods [11]. The rationale for isolation of postnatal day 7 DFSCs is our observation of Wnt5a expression in alveolar bone, ameloblasts and odontoblasts (Figure 1). Briefly, the dissected dental follicles were digested with 0.1% collagenase type I and 10 U/ml dispase (Sigma, St. Louis, MO, USA) for 1 hour at 37°C. The isolated DFSCs (Figure 1Q) were transferred to a T25 culture flask containing Dulbecco’s modified Eagle’s medium (DMEM, low glucose; Gibco, Grand Island, NY, USA) with 10% fetal bovine serum (Gibco) and 1% penicillin/streptomycin (Gibco). Upon 70 to 80% confluence, DFSCs were cultured to no more than four passages and used in all experiments.

### Wnt5a overexpression

pCDH-CMV-WNT5a-EF1-copGFP was constructed by inserting Wnt5a cDNA into a lentiviral vector (pCDH-CMV-MCS-EF1-copGFP; System Bioscience, Mountain View, CA, USA) as per our prior methods [11]. The cloned plasmid, psPAX and pMD2.G were transfected in a 4:3:1 proportion for virus packaging [14]. Green fluorescent protein (GFP)-positive cells were selected by fluorescence-activated cell sorting (FACS). Nontransfected and transfected cells with ~90% confluence were digested by trypsin, centrifuged (244 × *g*, 4 minutes) and resuspended with a density of 3.0 × 10^6^/ml. A total of 100 μl cell resuspension solution was added to each tube, centrifuged at 244 × *g* for 4 minutes and resuspended with 300 μl buffer before FACS (FACS Calibur; BD, Becton NJ, USA). Transfection efficiency was confirmed by quantitative RT-PCR (Taqman).

### Osteogenic differentiation

DFSCs at a density of 1 × 10^5^ cells per well (12-well plate) were exposed to DMEM, 10% fetal bovine serum, 10 mmol/l β-glycerophosphate, 50 μm/l ascorbate-2-phosphate and 0.1 μm/l dexamethasone (Sigma). Alizarin red was used to visualize mineral deposition. Cells were cultured in 12-well plates and used for osteogenic differentiation with a cell density of 1 × 10^5^ per well. For alizarin red staining, cells were fixed in 95% ethanol for 10 minutes, and exposed to 0.1% alizarin red for 30 minutes. Images were taken under the same camera parameters and processed into Adobe Photoshop under the same conditions (Adobe Systems Incorporated, San Jose, CA, USA). The alizarin red staining area was selected with the color range selection command using the same median threshold. The alizarin red area ratio was calculated by dividing the selected pixels over the total pixels area. Wnt5a protein (300 ng/ml; R&D Systems, Minneapolis, MN, USA) was added to osteogenesis medium or DMEM for osteogenic induction.

### Cell counting

GFP^+^ DFSCs or Wnt5a^+^/GFP^+^ DFSCs were seeded in 96-well plates at 1 × 10^4^ cells per well. Following overnight incubation, cells were treated with 10 μl Cell Counting Kit 8 (Dojindo, Rockville, MD, USA) at 1, 2, 3, 4 or 5 days. Absorbance at 450 nm was measured in triplicate with a microplate reader (Tecan, Mannedorf, Switzerland) following 1-hour incubation.

### Cell migration

The cells were first transfected with lentiviral GFP and then selected by FACS to obtain a high yield of GFP^+^ cells. A total of 5 × 10^4^ GFP^+^ DFSCs and Wnt5a^+^/GFP^+^ DFSCs in 100 μl DMEM were loaded into 8 μm pore Transwells (Corning, Corning, NY, USA) in 24-well plates as per our prior methods [15]. Following 12-hour incubation, migrated cells were trypsinized and counted, as per our prior methods [11].

### Western blot

GFP^+^ DFSCs or Wnt5a^+^/GFP^+^ DFSCs were plated at 1 × 10^6^ cells per well in six-well plates. Total proteins were extracted using RIPA buffer as per the manufacturer’s protocol. Primary antibodies included anti-Wnt5a (1:500; Abcam), anti-Runt-Related Transcription Factor 2 (Runx2, 1:500; Santa Cruz, Dallas, TX, USA), anti-osteocalcin (Ocn, 1:500; Santa Cruz, NM, Dallas, TX, USA) and anti-alkaline phosphatase antibody (ALP, 1:500; Abcam), with anti-glyceraldehyde-3-phosphate dehydrogenase (1:3,000; Abcam) as control. All assays were performed in triplicate.

### Quantitative RT-PCR (Taqman)

Total RNA was extracted using Trizol (Invitrogen, Grand Island, NY, USA) from GFP^+^ DFSCs and Wnt5a^+^/GFP^+^ DFSCs, and treated with RNase-free DNase. A total of 2 μg RNA per sample was used for cDNA synthesis primed with random hexamers. For PCR amplification, initial amplification using gene-specific primers was performed with denaturation at 95°C for 3 minutes, followed by 39 cycles at 95°C for 10 seconds, primer annealing at 55°C for 10 seconds, and primer extension at 72°C for 30 seconds. Quantitative, real-time PCR (Taqman) was used to determine fold mRNA differences relative to the control, and normalized to glyceraldehyde-3-phosphate dehydrogenase. Primer sequences were as follows (Invitrogen): Alp, ATGCCCTGAAACTCCAAA and CTCCAGCCGTGTCTCCTC; OCN, AGCAGGAGGGCAGTAAGG and TCCAGGGGATCTGGGTAG; collagen type 1 (Col1a1), ATTCACCTACAGCACGCTT and GGAGGTCTTGGTGGTTTT; and Runx2, TAGAGGGGATGCCTTAGTG and GAGGATGGAGGGAAACAA. Receptor activators for nuclear factor-κB ligand (RANKL) and osteoprotegerin (OPG) were purchased from Applied Biosystems, Inc. (catalogue number 4331182; Grand Island, NY, USA).

### Statistical analysis

Upon confirmation of normal data distribution, all quantitative datasets were subjected to Student *t* tests or one-way analysis of variance with *P* <0.05 for statistical significance.

## Results

Immunohistochemistry showed Wnt5a expression in postnatal tooth germ from 1 to 11 days. At day 1, Wnt5a was primarily expressed in alveolar bone (Figure 1B) in comparison with the control (Figure 1A). By day 3, Wnt5a became robustly expressed but was still restricted to the alveolar bone (Figure 1D) with little expression in either ameloblasts or dental papilla relative to the control (Figure 1C). Remarkably, Wnt5a showed robust expression in the odontoblast layer of the dental papilla by day 5, while remaining expressed in alveolar bone (Figure 1F), relative to the control (Figure 1E). By day 7, both ameloblasts and odontoblasts showed remarkable Wnt5a expression (Figure 1H) relative to the control (Figure 1G), whereas it was still expressed in alveolar bone. Wnt5a expression in ameloblasts and odontoblasts as well as the alveolar bone persisted at days 9 and 11 (Figure 1J,L), relative to controls (Figure 1I,K). By day 11, Wnt5a expression was mostly in ameloblasts and alveolar bone (Figure 1L), relative to the control (Figure 1K). Strikingly, there was little Wnt5a expression in dental pulp on the tested postnatal days 1 to 11 except for the odontoblast layer of dental papilla at postnatal days 5, 7, 9 and 11 (Figure 1F,H,J,L). We then isolated DFSCs from the first molar of postnatal day 7 rat mandible, as shown on the buccal (Figure 1M) or lingual (Figure 1N) side. The isolated first molar with dental follicle is shown in Figure 1O. Dental follicle was isolated from the first molar (Figure 1P). The isolated DFSCs were plated and assumed typical fibroblast-like morphology (Figure 1Q). Wnt5a protein was expressed in the dental follicle (Figure 1D,H,J,L), although it was not nearly as robust as in the adjacent alveolar bone.

**Figure 2:**
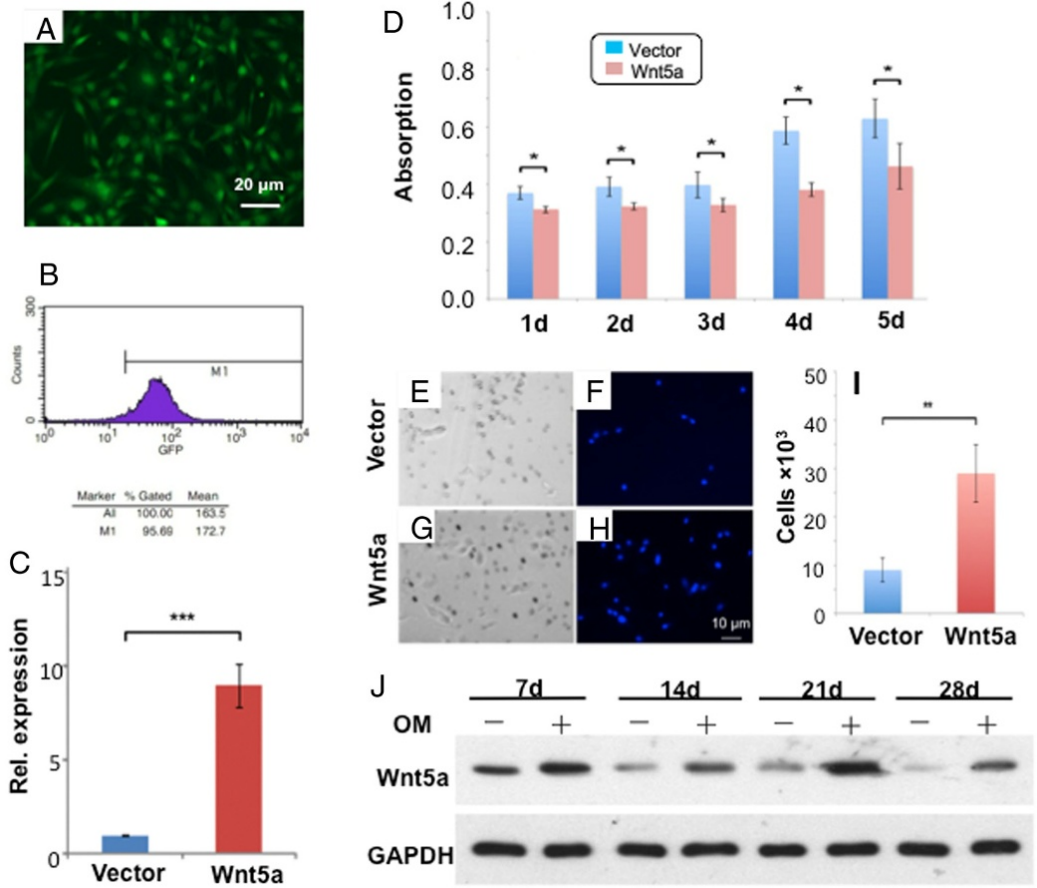
**Wnt5a overexpression and dental follicle stem/progenitor cell proliferation and migration. (A)** Transfection of lentiviral green fluorescence protein (GFP)-Wnt5a in dental follicle stem/progenitor cells. **(B)** GFP^+^ (Wnt5a^+^) cells were sorted by fluorescence-activated cell sorting (FACS), yielding >96% cells with positive GFP signal. **(C)** Real-time quantitative PCR (Taqman) showing Wnt5a overexpression (*n* = 3; *P* <0.001). **(D)** Cell Counting Kit 8 assay was applied to evaluate the influence of Wnt5a on cell proliferation. Proliferation rates of dental follicle stem/progenitor cells were attenuated upon Wnt5a overexpression (*n* = 3; *P <0.05). **(E, F)** Wnt5a-free, vector control dental follicle cells migrated through Transwell pores. **(G, H)** Wnt5a-overexpressed dental follicle cells showing robust migration through Transwell pores. **(I)** Quantitatively, ~3× more Wnt5a-overexpressed cells migrated than the control (*n* = 3; **P <0.01). **(J)** Native dental follicle cells with enhanced Wnt5a expression when exposed to osteogenesis induction medium (OM) for 7, 14, 21 and 28 days by western blot. GAPDH, glyceraldehyde-3-phosphate dehydrogenase.

Wnt5a was then overexpressed in postnatal day 7 DFSCs by GFP lentivirus (Figure 2A), and then GFP^+^ (Wnt5a^+^) cells were selected by FACS (Figure 2B). Wnt5a expression in lentiviral GFP transfected cells was confirmed relative to vector control (Figure 2C). Wild-type dental follicle cells proliferated in the observed 5 days in culture (Figure 2D). However, a subpopulation of Wnt5a overexpressed dental follicle cells showed significantly attenuated proliferation at each of the observed 5 days (Figure 2D). Migration of dental follicle cells is of interest not only for their putative ability to constitute multiple tissues of the periodontium, but also in their recruitment in wound healing [12, 13, 16]. Accordingly, we performed a Transwell assay to appreciate the migration capacity of vector control and Wn5a-overexpressed DFSCs. Relative to vector control (Figure 2E,F), Wnt5a-overexpressed dental follicle cells showed robust migratory capacity (Figure 2G,H). Significantly more Wnt5a transfected dental follicle cells migrated than vector control (Figure 2I). To begin to appreciate the roles of Wnt5a in differentiation, we exposed native dental follicle cells to osteogenesis induction medium for 7, 14, 21 and 28 days and found consistently enhanced Wnt5a expression (Figure 2J).

**Figure 3:**
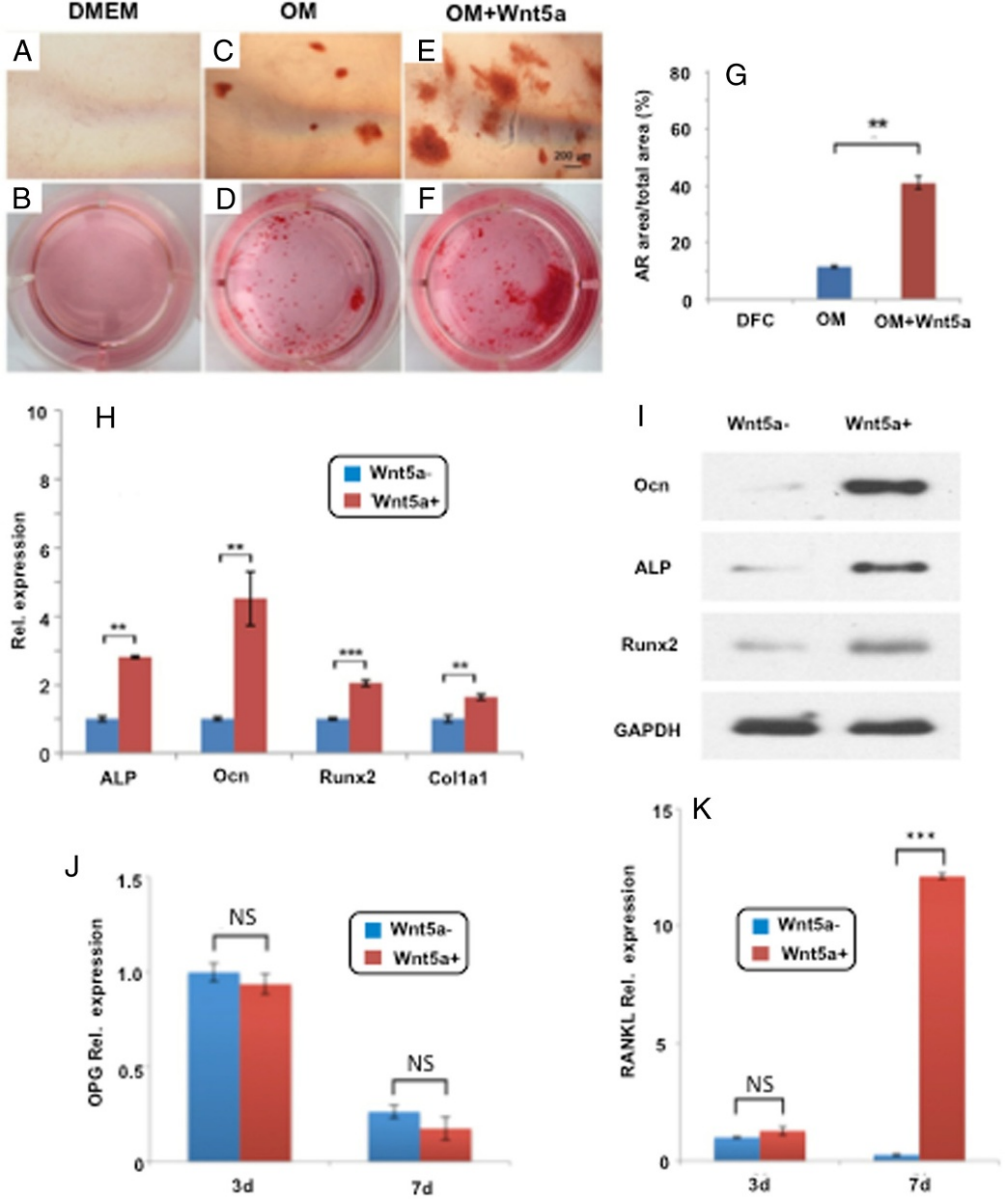
**Dental follicle stem/progenitor cell differentiation upon Wnt5a protein exposure. (A, B)** Dental follicle stem/progenitor cells showed virtually no alizarin red area upon exposure to Dulbecco’s modified Eagle’s medium (DMEM) without osteogenic supplements for the tested 14 days. **(C, D)** Dental follicle stem/progenitor cells exposed to osteogenic medium (OM) yielded a moderate alizarin red area. **(E, F)** Dental follicle stem/progenitor cells upon exposure to both Wnt5a protein (300 ng/ml) and osteogenic medium generated some alizarin red area. **(G)** Quantitatively, dental follicle stem/progenitor cells upon exposure to Wnt5a protein and osteogenic medium showed significantly greater alizarin red (AR) area than Wnt5a exposure alone, which in turn was more significant than wild-type dental follicle cells (DFC; *n* = 3; **P <0.01). **(H)** Real-time quantitative PCR (Taqman) revealed alkaline phosphatase (ALP), osteocalcin (Ocn), Runx2 and Col1a1 mRNA expression upon Wnt5a protein exposure for 7 days (*n* = 3; **P <0.01). **(I)** ALP, Ocn and Runx2 protein expression as treated with Wnt5a protein for 7 days. **(J, K)** Osteoprotegerin (OPG) and receptor activator for nuclear factor-κB ligand (RANKL) mRNA expression when dental follicle stem/progenitor cells were exposed to Wnt5a protein for 3 and 7 days. GAPDH, glyceraldehyde-3-phosphate dehydrogenase; NS, not significant; ***(*P* <0.01).

We further explored whether Wnt5a was capable of inducing dental follicle cell differentiation. Interestingly, native dental follicle cells failed to undergo spontaneous mineralization during the tested 14-day culture in DMEM (Figure 3A,B). Even in chemically defined, osteogenesis induction medium, native dental follicle cells only showed modest mineralization (Figure 3C,D). Upon exposure to 300 ng/ml Wnt5a protein in osteogenesis induction medium, mineralization by dental follicle cells was somewhat more pronounced, but remained unimpressive (Figure 3E,F). Quantitatively, Wnt5a protein addition in osteogenic medium yielded significantly larger alizarin red area than osteogenic medium alone, which in turn was significantly larger than native dental follicle cells without any osteogenic stimulation (Figure 3G). However, ~40% alizarin red area (Figure 3G) is far from overwhelming mineralization. Wnt5a-treated cells showed significantly higher expression of alkaline phosphatase, Ocn, Runx2 and Col1a1 (Figure 3H,I), again suggesting that native dental follicle cells may have moderate ability towards biomineralization. Chemically induced or Wnt5a-induced mineralization occurred sparsely in culture, with the majority of culture-plate remained unmineralized (Figure 3C,D,E,F), suggesting that large fractions of DFSCs may not readily mineralize even in the presence of chemically defined, osteogenesis medium or Wnt5a protein addition. Given this finding, we tested OPG and RANKL expression and found that, in contrast to a lack of significant differences in OPG expression between native and Wnt5a-treated cells (Figure 3J), RANKL expression was significantly augmented (~10-fold) in Wnt5a-treated cells (Figure 3K), suggesting putative regulatory roles of the monocyte/osteoclast lineage and potential involvement in alveolar bone remodeling by dental follicle cells.

**Figure 4:**
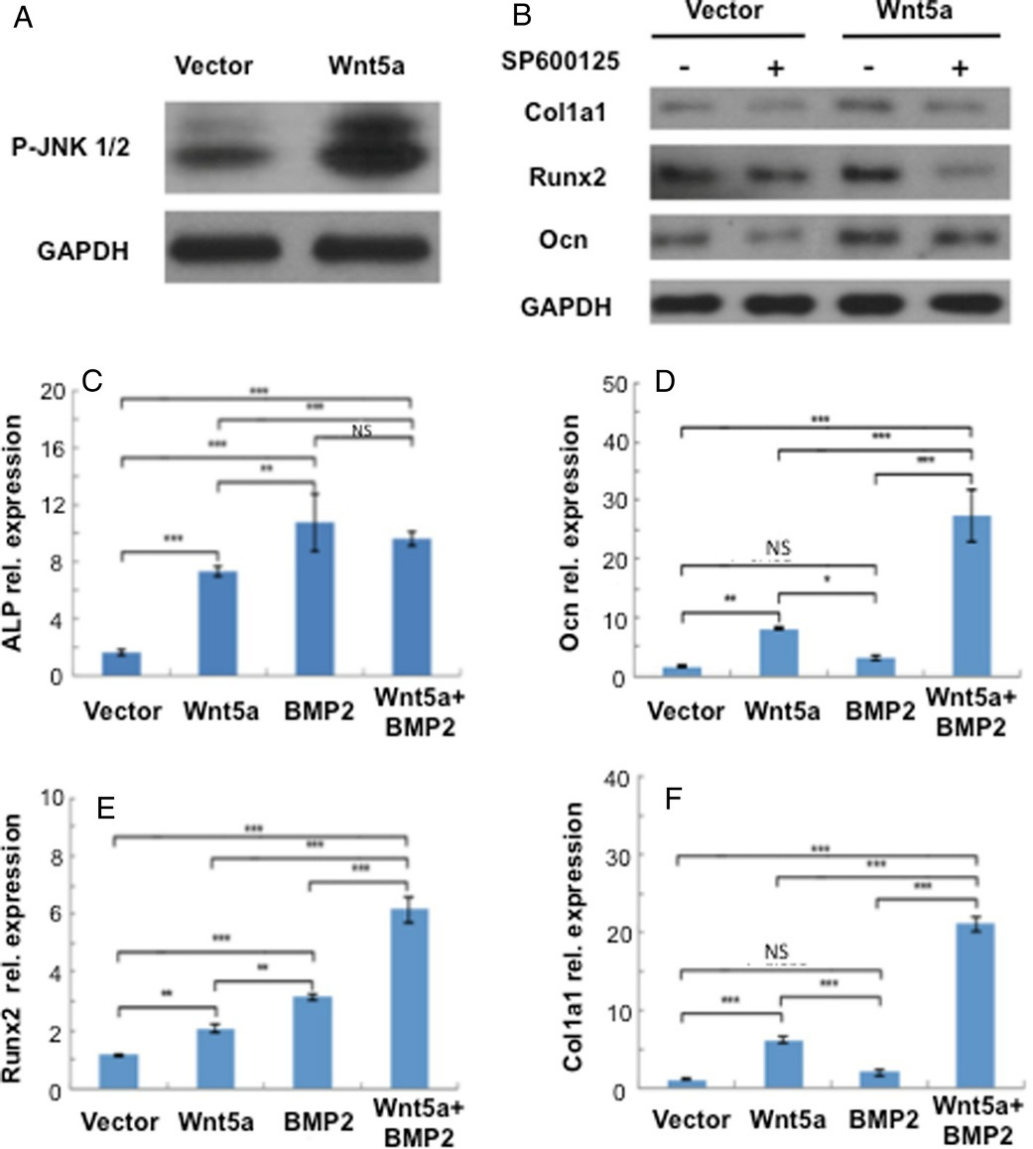
**Wnt5a signaling in dental follicle stem/progenitor cells. (A)** Western blot was used to detect phosphorylation-Jun N-terminal kinase (P-Jnk) 1/2 expression of Wnt5a transfected cells and vector control; higher expression of P-Jnk1/2 was observed in Wnt5a transfected cells. **(B)** Exposure to SP600125 for 3 days, a Jnk inhibitor, attenuated Col1a1, Runx2 and osteocalcin (Ocn) in Wnt5a transfected dental follicle cells relative to vector controls as demonstrated by western blot. **(C, D, E, F)** Real-time quantitative PCR (Taqman) revealed synergistic effects of Wnt5a when exposed to 100 ng/ml BMP2 for 3 days by upregulating Ocn, Runx2 and Col1a1 mRNA, but not alkaline phosphatase (ALP) activity (*n* = 3; **P <0.01; ***P <0.001). GAPDH, glyceraldehyde-3-phosphate dehydrogenase; NS, not significant.

We then probed Wnt5a signaling pathways in DFSCs. Wnt5a transfected cells showed robust phosphorylation-Jun N-terminal kinase (P-Jnk) 1/2 expression in comparison with vector control in 3 days (Figure 4A). Upon exposure to SP600125 for 3 days, a Jnk inhibitor, Col1a1, Runx2 and Ocn mRNAs were attenuated in Wnt5a transfected DFSCs (Figure 4B). In the presence of 100 ng/ml bone morphogenetic protein (BMP) 2, Wnt5a prompted dental follicle cells to upregulated Ocn (Figure 4D), Runx2 (Figure 4E) and Col1a1 (Figure 4F), in comparison with Wnt5a overexpression (Figure 4D,E,F) or Wnt5a alone (Figure 3H,I). However, ALP activity showed a significant decrease upon combined Wnt5a and BMP2 treatment (Figure 4C).

## Discussion

These findings provide the first glimpse of Wnt5a expression and putative functions in DFSCs in postnatal periodontium. Given robust Wnt5a expression in the alveolar bone on the observed postnatal days 1 to 11, we initially assumed that the isolated postnatal day 7 DFSCs would undergo robust mineralization. Contrarily, native DFSCs show somewhat modest mineralization even in osteogenesis induction medium or when exposed to Wnt5a protein. This is consistent with recent data showing a lack of enhanced bone formation or mineralization in a transgenic mouse model with Wnt5a overexpression [17]. Strikingly, our finding of ~10-fold enhanced RANKL expression in Wnt5a-treated DFSCs suggests that dental follicle may exert regulatory roles for monocyte/osteoclast lineages and potentially is involved in alveolar bone remodeling. Wnt5a appears to mediate DFSCs to undergo limited mineralization, perhaps consistent with their multipotency towards differentiation into not only PDL cells that do not mineralize in homeostasis, but also cementoblasts and/or alveolar bone osteoblasts that readily mineralize. The present findings suggest that Wnt5a plays putative roles in the fate of DFSCs towards differentiation into unmineralizing PDL cells or mineralized cementoblasts and/or alveolar bone osteoblasts.

Wnt5a expression in postnatal periodontium is robust in the alveolar bone as well as in ameloblast and odontoblast layers. In the embryonic tooth germ, Wnt5a expression is primarily confined to dental mesenchyme [3]. By embryonic day 14.5, however, Wnt5a is expressed in the enamel knot [3]. Therefore, our observed postnatal Wnt5a expression in the developing ameloblasts and odontoblasts appears to be a continuation of the presence of Wnt5a in prenatal tooth germ. The Wnt5a expression in alveolar bone is remarkable. Accordingly, we isolated DFSCs and found that they are somewhat modest in their innate ability to differentiate into osteoblasts, in comparison with postnatal bone marrow stromal cells that readily differentiate into osteoblasts *ex vivo* under permissive conditions [6]. A cautionary note is that *in vitro* assays of osteogenesis including alizarin red and von Kossa depend on culture medium conditions [18]. Stronger Wnt5a expression when native DFSCs are exposed to osteogenesis induction medium suggests its involvement in mineralization, perhaps towards cementoblasts and/or alveolar osteoblasts. In balance, however, osteogenesis-related genes including Ocn, Runx2 and collagen1a1 are upregulated only in the presence of both Wnt5a and BMP2, consistent with in prenatal dental follicle cells [19]. Our ongoing work explores the crosstalk between Wnt5a and BMP signaling in DFSCs. Our finding of RANKL expression upon Wnt5a treatment in DFSCs was motivated by their unimpressive osteogenesis, consistent with recent data showing that Wnt5a overexpression in a transgenic mouse model presented a lack of enhanced bone formation or mineralization [20]. RANKL upregulation by Wnt5a overexpression in DFSCs, but not OPG, is consistent with previous results that noncanonical signaling receptor Ror2 is expressed in osteoclast precursor cells and, by binding to Wnt5a, activates osteoclastogenesis [8, 21, 22]. Wnt5a plays putative roles in bone metabolism and bone remodeling – perhaps as a moderate osteogenesis enhancer, yet it may mediate osteoclastogenesis by RANKL – in ways that are important for maintaining the unmineralized PDL and mineralized cementum/alveolar bone. This speculation obviously requires additional investigations to prove or disapprove.

Wnt5a mediates noncanonical Wnt signaling and regulates cell proliferation, migration and polarization [23]. C-Jun N-terminal kinase (JNK) is a member of the mitogen-activated protein kinase family. When tyrosine and threonine are phosphorylated, JNK is activated [24]. Our finding of Wnt5a activation of the p-Jnk1/2 pathway, and conversely inhibition of p-Jnk1/2 by SP600125, indicates that noncanonical signaling is activated in DFSCs, similar to Wnt5a activation of intracellular c-Jun signaling in dental papilla cells [25] and bone marrow stromal cells [26]. JNK signaling is involved in cell polarization [27], migration [25] and osteogenic differentiation. Attenuation of Runx2, Ocn and Col1a1 expression upon application of JNK inhibitor SP600125 to dental follicle cells suggests that Wnt5a-mediated osteogenesis, counteracted by JNK inhibitors, is important for mineralization of dental follicle cells. Wnt5a overexpression attenuates the proliferation of DFSCs, and probably predisposes them for differentiation, consistent with previous reports of Wnt5a effects in other cell types [28]. Another fundamental cell behavior is migration, which is important for both development and tissue regeneration [12, 13, 15, 29]. Wnt5a clearly enhances the migration of DFSCs as we discovered here, consistent with Wnt5a promotion of tumor cell migration [30–32].

## Conclusions

Wnt5a appears to play important roles in the fate of DFSCs in development, homeostasis and perhaps regeneration of the periodontium, with potential implications in tooth eruption, orthodontic tooth movement, dental implant bone healing and periodontal disease.

## Abbreviations

ALP: alkaline phosphate
BMP: bone morphogenetic protein
Col1a1: collagen type I
DFSC: dental follicle stem/progenitor cell
DMEM: Dulbecco’s modified Eagle’s medium
FACS: fluorescence-activated cell sorter
GFP: green fluorescent protein
JNK: Jun N-terminal kinase
Ocn: osteocalcin
OPG: osteoprotegerin
PDL: periodontal ligament
P-Jnk: phosphorylation-Jun N-terminal kinase
RANKL: receptor activator for nuclear factor-κB ligand
Ror: tyrosine kinase-like orphan receptor
Runx2: Runt-related transcription factor 2.
